# Multidisciplinary Care for Older People With HIV: Optimizing Medication and Enhancing Quality of Life

**DOI:** 10.1093/ofid/ofag378

**Published:** 2026-06-22

**Authors:** Fatma Nisa Balli Turhan, Emre Kara, Zeynep Şahiner, Cafer Balci, Meliha Çağla Sönmezer, Ahmet Çağkan Inkaya, Meltem Gülhan Halil, Serhat Ünal, Kutay Demirkan

**Affiliations:** Department of Clinical Pharmacy, Gazi University Faculty of Pharmacy, Ankara, Türkiye; Department of Clinical Pharmacy, Hacettepe University Faculty of Pharmacy, Ankara, Türkiye; Department of Clinical Pharmacy, Hacettepe University Faculty of Pharmacy, Ankara, Türkiye; Division of Geriatrics, Department of Internal Medicine, Hacettepe University Faculty of Medicine, Ankara, Türkiye; Division of Geriatrics, Department of Internal Medicine, Hacettepe University Faculty of Medicine, Ankara, Türkiye; Department of Infectious Diseases and Clinical Microbiology, Hacettepe University Faculty of Medicine, Ankara, Türkiye; Department of Infectious Diseases and Clinical Microbiology, Hacettepe University Faculty of Medicine, Ankara, Türkiye; Division of Geriatrics, Department of Internal Medicine, Hacettepe University Faculty of Medicine, Ankara, Türkiye; Department of Infectious Diseases and Clinical Microbiology, Hacettepe University Faculty of Medicine, Ankara, Türkiye; Department of Clinical Pharmacy, Hacettepe University Faculty of Pharmacy, Ankara, Türkiye

**Keywords:** aging, HIV, multidisciplinary team, potentially inappropriate prescribing, quality of life

## Abstract

**Background:**

The growing number of older people with HIV (PWH) presents challenges in comprehensive care and maintaining health-related quality of life (HRQoL). This study aimed to evaluate the effect of a multidisciplinary intervention focusing on medication optimization and person-centered support on the HRQoL of older PWH.

**Methods:**

This prospective study included 3 groups: PWH ≥ 50 years (older); PWH < 50 years (younger); and HIV-negative individuals ≥ 50 years. Older PWH underwent a comprehensive assessment at baseline and approximately 6 months later, with interventions provided by clinical pharmacists, infectious disease specialists, and geriatricians. Medication Regimen Complexity Index (MRCI), STOPP/START and TIME criteria, and 36-Item Short Form Health Survey (SF-36) were used.

**Results:**

A total of 285 participants were included in this study (50% older PWH, 25% younger PWH, and 25% HIV-negative individuals). The mental component summary score of SF-36 was significantly higher in older PWH compared to younger PWH at baseline (*P* = .001). Antiretroviral therapy regimens were simplified in 24.1%, and at least 1 medication was deprescribed in 48.9% of older PWH. Older PWH demonstrated significant reductions in potentially inappropriate prescribing with STOPP/START and TIME criteria (*P* < .001) at the second interview, but no significant changes were observed in MRCI scores (*P* > .05). Additionally, physical and mental component summary scores of SF-36 increased significantly (*P* = .012 and *P* < .001, respectively) after intervention.

**Conclusions:**

Multidisciplinary care was associated over time with reductions in inappropriate prescribing and improvements in HRQoL among older PWH. These findings underscore the need for integrated, person-centered, age-tailored HIV care models to support healthy aging.

## Teaser

A multidisciplinary, person-centered intervention was associated with improvements in health-related quality of life and reductions in inappropriate prescribing among older people with HIV, highlighting the potential value of integrated, age-tailored HIV care models to optimize pharmacotherapy and support healthy aging.

## 1. INTRODUCTION

Recent innovations in antiretroviral treatment (ART) have transformed HIV infection from a rapidly progressive, fatal condition into a stable chronic disease requiring ongoing medical management. The proportion of older people with HIV (PWH), particularly those aged 50 years and above, has steadily risen, presenting new challenges in clinical care for this aging population [1].

Prolonged HIV infection, in combination with the long-term effects of ART and chronic inflammation, contributes to a higher prevalence of comorbidities at a younger age compared with the general population [2]. However, it is known that this early comorbidity profile may be significantly influenced by non-HIV factors—including lifestyle, behavioral, and socioeconomic determinants—which may contribute as much as HIV-specific factors to these conditions [1, 2]. The coexistence of comorbidities in older PWH requires co-medications, which increases the medication burden. Although modern combination of ART in 1 pill has reduced the daily medication burden of antiretroviral medications, the addition of co-medications increases the risk of polypharmacy, leading to a greater overall medication burden compared with HIV-negative peers [3]. This burden is associated with drug interactions, potentially inappropriate prescribing (PIP), adverse events, nonadherence, and hospitalization [4, 5].

Even when virologically and immunologically stable, older PWH have a lower health-related quality of life (HRQoL) compared to the general population [6]. Beyond HIV infection, factors such as aging, stigma, multimorbidity, and polypharmacy further reduce HRQoL [7]. As this population continues to grow and age, there is a need to shift the focus from virologic suppression alone toward improving overall health and HRQoL [8]. Accordingly, incorporating HRQoL assessment alongside traditional clinical endpoints, such as viral load and CD4+ T-cell count, is increasingly recommended when evaluating therapeutic strategies for PWH [9]. Given their complex health needs, improving HRQoL requires multidisciplinary care, involving geriatricians to address age-related syndromes and multimorbidity, and pharmacists to optimize medication use and mitigate polypharmacy [5, 10, 11].

Health-related quality of life is widely recognized as a crucial component of HIV care, yet few studies have evaluated interventions to improve HRQoL in the general PWH population [12]. Moreover, although medication optimization is regarded as a key strategy to reduce polypharmacy and support overall well-being, its specific impact on HRQoL among older PWH remains unclear. This gap highlights the need to evaluate multidisciplinary approaches that integrate medication review with comprehensive care for this population. Therefore, the primary aim of this study was to improve HRQoL in PWH aged ≥50 years through medication optimization, with a secondary aim of comparing their health outcomes with those of HIV-negative individuals aged ≥50 years and younger PWH.

## 2. MATERIALS AND METHODS

### 2.1. Study Design and Population

This prospective study was conducted between February 2022 and December 2023, with protocol approval from the Local Ethics Committee (GO 22/73). It included 3 groups: PWH aged ≥50 years (older PWH), PWH aged <50 years (younger PWH), and HIV-negative individuals aged ≥50 years. The age threshold of ≥50 years was selected based on HIV-related literature, where PWH aged ≥50 years were commonly described as older adults due to the early onset of age-related comorbidities [2]. Participants were recruited from 2 centers: PWH from a tertiary care university hospital and HIV-negative individuals from an outpatient family health center affiliated with the study institution (Hacettepe University) and run by primary care physicians. These individuals were recruited among adults aged ≥50 years attending routine primary care visits (eg, prescription renewal) without acute complaints at the time of assessment, to better represent a community-based population.

Inclusion criteria for PWH were current use of ART and an undetectable viral load for at least 3 months. Younger PWH were additionally required to be aged ≥18 years. Older PWH who met the inclusion criteria were prospectively assessed at 2 time points: baseline (first interview) and at their first hospital visit 6 months later (second interview). Younger PWH and HIV-negative individuals were assessed only at baseline. Among older PWH, participants who did not attend the follow-up visit were considered lost to follow-up and excluded from the final analysis.

### 2.2. Measures

**Demographic and clinical characteristics—**Demographic data, clinical characteristics, comorbidities, and detailed information on medications, including dosage, timing, and route of administration, were obtained through the hospital's electronic medical record systems and direct verbal communication with participants.

**Medication count and complexity—**Medication count was assessed in 2 ways: by the number of active substances and by the total number of pills taken. Polypharmacy was defined as the use of 5 or more different medications [13].

The complexity of each participant's medication regimen was evaluated using the Turkish-validated version of the Medication Regimen Complexity Index (MRCI). This index quantifies treatment complexity by evaluating 3 domains: pharmaceutical form (section A), dosing frequency (section B), and administration instructions (section C). A higher total MRCI score indicates a more complex treatment regimen [14].

**PIP—**The potentially inappropriate medications (PIMs) and potential prescribing omissions (PPOs) among older PWH were classified according to the Screening Tool of Older People's Prescriptions and the Screening Tool to Alert to Right Treatment (STOPP/START, version 2) [15], as well as the Turkish Inappropriate Medication use in the Elderly (TIME) criteria [16].

**Comprehensive geriatric assessment (CGA)—**The CGA was performed in all participants aged ≥50 years using tools that have been validated and demonstrated reliability in the Turkish population. Functional dependency was assessed using the Katz Activities of Daily Living (ADL) scale [17], with higher scores indicating greater independence. Nutritional status was evaluated using the Mini Nutritional Assessment–Short Form (MNA-SF; 0–14 points), with scores ≥12 considered normal [18]. Depressive symptoms were detected using the 15-item Yesavage Geriatric Depression Scale (YGDS) [19]. Sarcopenia risk was evaluated with the SARC-F questionnaire, covering strength, assistance in walking, rising from a chair, climbing stairs, and history of falls, where a score of ≥4 indicates sarcopenia risk [20]. Cognitive function was assessed using the Mini Mental State Examination (MMSE), with scores ≤24 indicating cognitive impairment [21]. Frailty was evaluated with both the FRAIL scale [22], which assesses fatigue, resistance, ambulation, illnesses, and weight loss, and the Clinical Frailty Scale [23].

**HRQoL—**HRQoL was assessed using the Turkish-validated version of the 36-Item Short Form Survey (SF-36). The SF-36 includes 2 summary scores: the physical component summary (PCS) and the mental component summary (MCS), both scored from 0 to 100, with higher scores indicating better HRQoL. It also comprises 8 subscales: physical functioning, role-physical, general health, and pain, which contribute to the PCS, and social functioning, role-emotional, vitality, and mental health, which contribute to the MCS [24].

Comprehensive geriatric assessments were performed by experienced geriatricians, while HRQoL instruments were administered by clinical pharmacists with postgraduate training and experience in geriatric patient assessment. To ensure standardization and minimize interassessor variability, all assessments were conducted by the same individuals using validated instruments and consistent procedures.

The primary outcome of the study was the change in PCS score. Secondary outcomes included changes in MCS and subscale scores, as well as number of PIMs, and PPOs identified using STOPP/START and TIME criteria.

### 2.3. Interventions

A multidisciplinary intervention, involving clinical pharmacists, infectious diseases specialists, and geriatricians, was applied only to older PWH. The intervention was designed to optimize pharmacotherapy and promote healthy aging. This approach was informed by findings from a standardized first interview, utilizing validated scales and criteria to identify the treatment needs and drug-related problems (DRPs) of older PWH. The components of the multidisciplinary intervention, including its structure, professionals involved, timing, and objectives, were summarized in Table 1. Clinical pharmacists conducted comprehensive medication reviews and provided structured recommendations to physicians and/or patients to optimize medication use, including simplifying ART regimens, deprescribing PIMs, or addressing PPOs. These recommendations were discussed with the attending physician and/or patient, and their acceptance was recorded. Drug-related problems and corresponding recommendations were classified according to the Pharmaceutical Care Network Europe (PCNE) Classification for Drug-Related Problems Version 9.1. Clinical decisions were made collaboratively by the multidisciplinary team based on explicit criteria, including STOPP/START and TIME criteria, current HIV treatment guidelines, and individual patient characteristics. Comprehensive geriatric assessment findings guided tailored interventions and indicated when further evaluation or specialist referral was required. The intervention was repeated at 6 months to evaluate treatment outcomes and changes in HRQoL. Both interviews were conducted in an outpatient setting, and each interview lasted approximately 60 minutes.

**Table 1.** Components of the Multidisciplinary Intervention

Component	Delivered by	Description	Timing	Objective
Medication review	Clinical pharmacist	Review using STOPP/START, TIME criteria, and current HIV guidelines	Baseline and follow-up	Identify PIPs
Comprehensive geriatric assessment	Geriatrician	Assessment of geriatric syndromes using standardized tools	Baseline	Individualized intervention planning
ART modification	Infectious diseases specialist	Optimization or simplification of ART according to current HIV guidelines and patient characteristics	Baseline: as needed	Improve adherence and reduce ART burden
Medication optimization	All team members	Discontinuation, dose reduction, or initiation of medications based on clinical indication, STOPP/START and TIME criteria, and multidisciplinary team recommendations	Baseline: as needed	Reduce PIPs
Patient education	All team members	Counseling on adherence and medication use	Baseline and follow-up	Improve adherence

Abbreviations: ART, antiretroviral treatment; PIPs, potentially inappropriate prescribing; STOPP/START, Screening Tool of Older People's Prescriptions and the Screening Tool to Alert to Right Treatment; TIME, Turkish Inappropriate Medication use in the Elderly.

### 2.4. Statistical Analyses

Sample size estimation was based on previously published data on SF-36 scores in PWH from Moore et al [25]. Two separate calculations were performed for the PCS and MCS, based on expected mean differences considered clinically meaningful in SF-36 scores. Assuming a 2-sided significance level (α) of 0.05, a statistical power of 95%, and variability consistent with the reported standard deviations in the reference study, the required sample size was estimated as 62 participants per group for PCS and 69 participants per group for MCS. The largest calculated sample size was therefore considered, and a minimum of 70 participants per group was targeted to ensure adequate statistical power for both outcomes. Sample size calculations were performed using G*Power 3.1 software.

Statistical analyses were performed using SPSS (version 23.0, IBM Corp., Chicago, IL, USA) for MacOS. Continuous variables were assessed for normality using visual and analytical methods. Normally distributed variables were expressed as mean ± SD, while nonnormally distributed variables were presented as median and first–third quartiles (Q1–Q3). Categorical variables were summarized as frequencies and percentages. For baseline comparisons between independent groups, the chi-square test was used for categorical variables. Continuous variables were compared using the Student's t-test or 1-way ANOVA for normally distributed data, and the Mann–Whitney U test or Kruskal–Wallis test for nonnormally distributed data, as appropriate. For within-group comparisons in older PWH (baseline vs 6-month follow-up), paired samples t-test was used for normally distributed variables, and the Wilcoxon signed-rank test was used for nonnormally distributed variables.

Physical component summary and MCS were calculated using Turkish normative data [26]. Changes in HRQoL were evaluated both at the population level and within individuals. In addition to statistical significance testing, clinically meaningful improvement in HRQoL was defined as a minimum clinically important difference (MCID) of 5 points in the PCS and MCS scores [27]. A *P* value of <.05 was considered statistically significant.

## 3. RESULTS

### 3.1. Patient Characteristics

**Figure 1.**
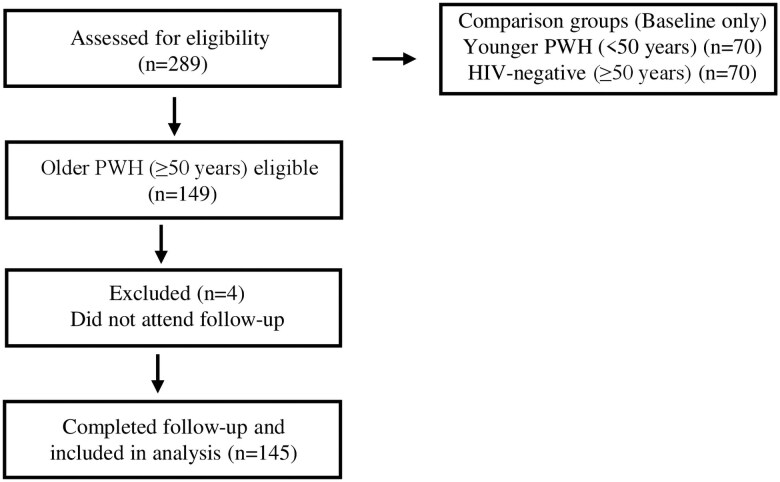
Flow diagram of study participants and follow-up. A total of 289 individuals were assessed for eligibility. Among these, 149 older people with HIV (PWH) met the inclusion criteria for follow-up. Four participants were excluded due to nonattendance at the follow-up visit and inability to be contacted. Consequently, 145 older PWH completed follow-up and were included in the final analysis. The comparison groups consisted of 70 younger PWH and 70 HIV-negative individuals, recruited to reach predefined sample sizes and assessed at baseline only.

Participant flow is summarized in Figure 1. Of the 149 older PWH eligible for follow-up, 4 were excluded due to nonattendance at the follow-up visit, resulting in 145 older PWH (79.3% male) included in the final analysis. Among older PWH, 18.6% were aged ≥65 years. The comparison groups consisted of 70 younger PWH (88.6% male) and 70 HIV-negative individuals (41.4% male), recruited to meet predefined sample sizes (Table 2). HIV-negative individuals were significantly older than older PWH (*P* = .006).

**Table 2.** Demographic and Clinical Characteristics of the Study Groups

	Older PWH ≥50 Y (n = 145)	Younger PWH <50 Y (n = 70)	HIV-Negative ≥50 Y (n = 70)
Male, n (%)	115 (79.3)	62 (88.6)	29 (41.4)
Age (years), mean (SD)	58.4 (7.5)	34.5 (6.3)	61.2 (5.9)
BMI (kg/m^2^), mean (SD)	27.8 (4.4)	24.6 (3.5)	29.8 (5.3)
Educational level, n (%)			
≤Middle school	68 (46.9)	11 (15.7)	33 (47.1)
High school	23 (15.9)	13 (18.6)	22 (31.4)
≥University	54 (37.2)	46 (65.7)	15 (21.4)
Years since HIV diagnosis, median (Q1–Q3)	8 (5–13)	7 (4–9)	-
Polypharmacy, n (%)	78 (53.8)	8 (11.4)	36 (51.4)
Number of chronic diseases, median (Q1-Q3)	1 (0–3)	0 (0–1)	2 (1–3)
Comorbidities, n (%)			
Hypertension	59 (40.7)	3 (4.3)	54 (77.1)
Diabetes mellitus	29 (20.0)	2 (2.9)	31 (44.3)
Dyslipidemia	44 (30.3)	2 (2.9)	16 (22.9)
Coronary artery disease	23 (15.9)	0 (0)	16 (22.9)
Depression	16 (11.0)	9 (12.9)	4 (5.7)

Abbreviations: BMI, body mass index; HIV, human immunodeficiency virus; Q1–Q3, first–third quartiles; PWH, people with HIV; SD, standard deviation.

### 3.2. Antiretroviral Medication Count and Complexity

Through a collaborative approach involving clinical pharmacists and physicians, antiretroviral regimen modifications were made for 35 (24.1%) older PWH based on current medication use and clinical status. Of these, 21 were switched to a dual regimen of dolutegravir and lamivudine (DTG + 3TC); 13 to a single-tablet regimen containing bictegravir, emtricitabine, and tenofovir alafenamide (BIC/FTC/TAF); and 1 to a combination of DTG, FTC, and tenofovir disoproxil fumarate (TDF). The number of antiretroviral active substances significantly decreased at the second interview (*P* < .001), whereas the number of antiretroviral pills significantly increased (*P* = .019) (Table 3).

**Table 3.** Antiretroviral Medication Counts and MRCI Scores of PWH

	Older PWH ≥50 Y (n = 145)			Younger PWH <50 Y (n = 70)	
	First interview	Second interview	*P* value	First interview	*P* value^a^
Antiretrovirals, median (Q1–Q3)					
Number of active substances	3 (2–3)	2 (2–3)	**<**.**001**	3 (3–3)	.149
Number of pills	2 (1–3)	2 (1–3)	.**019**	1 (1–2)	.**008**
Antiretroviral MRCI, median (Q1–Q3)					
Section A	1 (1–1)	1 (1–1)	1.000	1 (1–1)	1.000
Section B	2 (1–2)	2 (1–2)	.513	1 (1–2)	.**014**
Section C	3 (2–4)	3 (2–4)	.**036**	2 (2–3)	.**007**
Overall	6 (4–7)	6 (4–7)	.086	4 (4–6)	.**008**

Abbreviations: Q1–Q3, first–third quartiles; MRCI, Medication Regimen Complexity Index; PWH, people with HIV. Statistically significant *P*-values (*P* < 0.05) are shown in bold.

^a^Difference between the antiretroviral medication counts and MRCI scores at the first interview.

### 3.3. Potentially Inappropriate Prescribing

Significant reductions were observed in the proportions of older PWH with at least 1 PIP: 31%–15% for STOPP, 83%–48% for START, 21%–10% for TIME-to-STOPP, and 84%–48% for TIME-to-START (*P* < .001 for all).

According to the STOPP criteria, a total of 54 and 24 PIMs were identified in older PWH at the first and second interviews, respectively. The most frequent PIM was “any drug prescribed without an evidence-based clinical indication,” such as proton pump inhibitors (PPIs) and aspirin, with 34 PIMs at the first interview and 23 at the second interview (Supplementary Data). Similarly, based on the TIME-to-STOPP criteria, 35 and 14 PIMs were identified at the first and second interviews, respectively. The most frequent PIM was “inappropriate use of PPIs due to polypharmacy,” with 22 PIMs at the first interview and 11 PIMs at the second interview (Supplementary Data).

According to the START and TIME-to-START criteria, PPOs were 187 and 188 at the first interview and 84 and 85 at the second interview, respectively. The most common PPOs identified by both the START and TIME-to-START criteria were annual influenza and pneumococcal vaccinations. Following the first assessment, 42 (38%) out of 110 older PWH eligible for influenza vaccination reported receiving the vaccine, as did 39 (71%) out of 55 older PWH eligible for pneumococcal vaccination. In addition to vaccination-related PPOs, other frequently identified omissions included lack of vitamin D supplementation in individuals with osteopenia (n = 9), absence of antihypertensive therapy when indicated (n = 9), and lack of statin therapy in patients with documented atherosclerotic coronary artery disease (n = 3).

### 3.4. Drug-Related Problems and Corresponding Recommendations

Drug-related problems were identified in 117 (80.7%) older PWH, with a total of 216 problems and a median of 1 (range 0–5) DRP per individual. A total of 329 recommendations were proposed to address these problems by the clinical pharmacist. The most common underlying causes of DRPs were untreated or insufficiently treated indications (n = 54), followed by food–drug interactions (n = 39). Recommendations were made at the physician level (24.3%), patient level (42%), and drug level (33.7%). Of these, 95.1% were accepted by physicians and/or patients. Following implementation, 81% of DRPs were fully resolved, 6% were partially resolved, and 13% remained unresolved.

### 3.5. Co-medication Count, Total Medication Count, and Their Complexity

Following the multidisciplinary intervention, at least 1 medication was deprescribed in 71 older PWH (48.9%). The most frequently discontinued antiretroviral medication was TDF (n = 17), while the most frequently modified co-medication was PPIs (n = 17; discontinued in 5 individuals, dose reduced in 12). At least 1 new medication was initiated in 61 older PWH (42%). The most frequently initiated antiretroviral medications were BIC (n = 13) and DTG (n = 12), while co-medications included lipid-lowering agents (n = 22; statins in 21 and ezetimibe in 1 person), antihypertensives (n = 13), vitamin D (n = 9), and antidiabetic agents (n = 5). The number of co-medications and the total medications used by older PWH, and their overall MRCI scores, were similar between the first and second interviews (*P* > .05 for all; see Table 4).

**Table 4.** Number of Co-medications and Overall Medications and MRCI Scores of Participants Aged ≥50 Years

	PWH ≥ 50 Y (n = 145)			HIV-Negative ≥ 50 Y (n = 70)	
	First interview	Second interview	*P* value	First interview	*P* value^a^
Co-medications, median (Q1–Q3)					
Number of active substances	2 (0–5)	2 (1–5)	.423	5 (3–7)	**<**.**001**
Number of pills	2 (0–4)	2 (1–4.75)	.400	4 (2–6)	**<**.**001**
Co-medications MRCI, median (Q1–Q3)					
Section A	1 (0–1)	1 (1–1)	.852	1 (1–1)	.**004**
Section B	2 (0–5)	2 (1–5)	.524	4 (2–5)	**<**.**001**
Section C	1 (0–4)	1 (0–3)	.943	3 (2–5)	**<**.**001**
Overall	5 (0–10)	5 (2–10)	.644	9 (5–13)	**<**.**001**
Total medications, median (Q1–Q3)				…	**…**
Number of active substances	5 (3–7.5)	4 (2–7)	.065	5 (3–7)	.187
Number of pills	4 (2–6)	5 (2–7)	.063	4 (2–6)	.918
Total medications MRCI, median (Q1–Q3)					
Section A	1 (1–1)	1 (1–1)	.740	1 (1–1)	.806
Section B	3 (2–6)	4 (2–6)	.448	4 (2–5)	.539
Section C	5 (3–6)	5 (3–7)	.634	3 (2–5)	**<**.**001**
Overall	10 (6–14)	10 (6.5–15)	.483	9 (5–13)	.099

Abbreviations: HIV, human immunodeficiency virus; Q1–Q3, first–third quartiles; MRCI, Medication Regimen Complexity Index; PWH, people with HIV. Statistically significant *P*-values (*P* < 0.05) are shown in bold.

^a^Difference between the co-medication counts, total medication counts, and MRCI scores at the first interview.

### 3.6. Comprehensive Geriatric Assessments

HIV-negative individuals had significantly better nutritional status (*P* = .028), although the proportion of participants with MNA-SF scores below 12 did not differ between groups (*P* = .221). Conversely, they exhibited a higher risk of sarcopenia (*P* = .001). Frailty scores assessed by the FRAIL scale were similar between the groups, whereas CFS scores were significantly higher in HIV-negative participants (*P* = .006) (Table 5).

**Table 5.** Baseline CGA Results in Participants Aged ≥50 Years

CGA	PWH ≥ 50 Y (n = 145)	HIV-Negative ≥ 50 Y (n = 70)	*P V*alue
	First interview	First interview	
Katz ADL, median (Q1–Q3)	6 (6–6)	6 (6–6)	.487
MNA-SF, median (Q1–Q3)	14 (13–14)	14 (14–4)	.**028**
MNA-SF < 12 points, n (%)	13 (9)	3 (4.3)	.221
GDS, median (Q1–Q3)	0 (0–3)	2 (0–3)	.132
SARC-F, median (Q1–Q3)	0 (0–0)	0 (0–1)	.**001**
MMSE, median (Q1–Q3)	30 (29–30)	30 (30–30)	.980
MMSE < 24 points, n (%)	5 (3.4)	1 (1.4)	.666
CFS, median (Q1–Q3)	3 (2–3)	3 (3–3)	.**006**
FRAIL, median (Q1–Q3)	0 (0–1)	0 (0–1)	.961

Abbreviations: ADL, activities of daily living; CFS, Clinical Frailty Scale; CGA, comprehensive geriatric assessments; GDS, Geriatric Depression Scale; HIV, human immunodeficiency virus; Q1–Q3, first–third quartiles; MMSE, Mini Mental State Examination; MNA-SF, Mini Nutritional Assessment—Short Form; PWH, people with HIV. Statistically significant *P*-values (*P* < 0.05) are shown in bold.

### 3.7. Health-Related Quality of Life

Health-related quality of life scores assessed by the SF-36 are presented in Table 6. At baseline, PCS scores were similar between older and younger PWH, whereas younger PWH had significantly lower MCS scores compared to older PWH (*P* < .001). Among older PWH, a statistically significant improvement was observed in both PCS and MCS scores after 6 months (*P* = .012 and *P* < .001, respectively). In older PWH, the median change in the SF-36 PCS score was 0.2 (Q1–Q3: −0.9–2.5), while the corresponding change in the mental MCS score was 3.0 (Q1–Q3: 0–6.2). Regarding clinically meaningful improvements (MCID ≥ 5 points), 12.4% of participants (n = 18) demonstrated an increase in PCS, while 34.5% (n = 50) showed an improvement in MCS.

**Table 6.** SF-36 HRQoL Scores of the Study Groups

SF-36 Subscales	Older PWH ≥50 Y (n = 145)			HIV-negative ≥50 Y (n = 70)		Younger PWH <50 Y (n = 70)	
Median, (Q1–Q3)	First interview	Second interview	*P* value	First interview	*P* ^a^ value	First interview	*P* ^b^ value
Physical functioning	90 (75–95)	90 (80–95)	**<**.**001**	80 (70–90)	.**022**	100 (90–100)	**<**.**001**
Role-physical	100 (62.5–100)	100 (75–100)	**<**.**001**	100 (75–100)	.895	100 (68.75–100)	.162
General health	70 (55–87.5)	80 (65–90)	**<**.**001**	70 (53.8–80)	.281	57.5 (45–80)	.**002**
Pain	90 (77.5–100)	90 (77.5–100)	**<**.**001**	80 (57.5–90)	.**003**	90 (67.5–100)	.339
PCS	50.8 (42.2–53.5)	50.9 (45.5–52.9)	.**012**	48.9 (43.5–52.2)	.101	50.8 (46.4–54.0)	.239
Social functioning	100 (75–100)	100 (87.5–100)	**<**.**001**	100 (100–100)	.**009**	81.25 (50–100)	.**002**
Role-emotional	100 (66.7–100)	100 (66.7–100)	**<**.**001**	100 (66.7–100)	.188	100 (33.3–100)	.**026**
Vitality	70 (50–80)	75 (60–85)	**<**.**001**	60 (50–71.25)	.**021**	60 (50–75)	.**004**
Mental health	76 (60–84)	80 (68–88)	**<**.**001**	72 (64–80)	.386	68 (48–80)	.**005**
MCS	49.8 (35.8–56.0)	53.5 (43.9–58.8)	**<**.**001**	48.7 (38.0–54.8)	.563	40.2 (23.1–52.2)	.**001**

Abbreviations: HIV, human immunodeficiency virus; Q1–Q3, first–third quartiles; MCS, mental component summary; PCS, physical component summary; PWH, people with HIV. Statistically significant *P*-values (*P* < 0.05) are shown in bold.

^a^Difference in SF-36 scores at the first interview among people aged ≥50 y.

^b^Difference in SF-36 scores at the first interview among PWH.

## 4. DISCUSSION

To our knowledge, this is the first study to implement a multidisciplinary, team-based intervention specifically targeting older PWH and to report relations with reductions in PIP and improvements in both physical and mental HRQoL. Antiretroviral treatment regimens were simplified and inappropriate medications deprescribed, though overall MRCI remained stable. The finding that younger PWH had lower mental HRQoL further underscores the need for lifelong, individualized strategies to sustain well-being across the aging trajectory of HIV care.

In this study, the prevalence of PIMs among older PWH was 31% according to the STOPP criteria and 20.7% according to the TIME criteria. The STOPP criteria have been recently updated, and most of the criteria used to identify PIM in our study have remained unchanged in the revised version [28]. The higher detection rate observed with the STOPP criteria may be attributed to their broader and more generalizable approach, whereas the TIME criteria offer more context-specific and individualized assessments. This indicates that using multiple tools may capture a broader range of prescribing issues. Although the prevalence identified by the STOPP criteria falls within the previously reported range of 30%–71% in this population, the overall PIM detection rate was relatively low in our cohort [5, 13, 29, 30]. In contrast to previous studies, which identified benzodiazepines as the most prevalent PIMs, no benzodiazepine use was observed in our cohort [13, 29, 30]. This may be attributed to the low prevalence of substance use, local prescribing practices, and prior deprescribing efforts implemented before the study. In our study, PPIs and aspirin used without clear indications were the leading PIMs, consistent with recent European AIDS Clinical Society (EACS) guideline recommendations to deprescribe these medications when risks outweigh benefits [31]. These findings underscore the value of systematic medication review and deprescribing strategies guided by validated criteria to minimize PIM exposure. Postintervention, the prevalence of PIM decreased (15.2% STOPP, 9.7% TIME), and nearly half (48.9%) of older PWH had at least 1 medication deprescribed. These results demonstrate that multidisciplinary approaches can optimize pharmacotherapy and support rational medication use. Although our deprescribing rate was lower than the 69% reported by McNicholl et al in PWH aged ≥65, differences may reflect variations in age, baseline medication burden, and PIM assessment criteria [5].

In this study, a high prevalence of PPOs was identified, with 83.4% of participants missing at least 1 recommended treatment, most commonly influenza and pneumococcal vaccinations, consistent with findings from the limited available studies [29, 30]. Although vaccination rates improved significantly following the interventions, influenza vaccination uptake remained lower than aimed, likely due to logistical barriers. Beyond vaccines, statin therapy was the most frequently omitted treatment among older PWH, consistent with recent evidence and guideline recommendations supporting statin use for primary cardiovascular prevention in this population [32, 33]. This finding is supported by our previous work, which demonstrated that a considerable proportion of infectious diseases physicians in Türkiye did not routinely apply guidelines or use cardiovascular risk calculators for dyslipidemia management in PWH, highlighting barriers to optimal statin prescribing [34]. Moreover, Turkish regulation limits statin reimbursement to people with atherosclerotic heart disease, diabetes, and patients with elevated LDL levels (LDL > 190 mg/dL). The results highlight the importance of regular review of pharmacotherapy and preventive care in older PWH and indicate the need for PIP assessment tools tailored to their clinical characteristics.

Antiretroviral treatment modifications reduced the number of antiretroviral agents but unintentionally increased pill burden due to the transition to the DTG + 3TC regimen, which lacked a single-tablet formulation in Türkiye during the study period. This led to more frequent dosing and complex administration instructions, consistent with literature indicating that integrase inhibitor–based regimens may increase regimen complexity [35]. In addition, the initiation of new co-medications and changes in administration instructions may have further contributed to regimen complexity. As a result, despite optimization efforts, overall MRCI scores did not significantly change. We believe that the recent approval and reimbursement of a single-tablet DTG + 3TC formulation in Türkiye will contribute to simplifying ART and reducing pill burden.

Several studies have reported higher MCS in older PWH than in younger, consistent with our findings [36, 37]. This may be explained by younger individuals' increased vulnerability to HIV-related stigma and limited coping experience. Although younger PWH had better physical functioning, their general health perception was lower. This aspect, which may reflect poorer mental well-being, could have attenuated their overall PCS scores and led to similar results across age groups [7, 25, 37]. The comparable PCS scores across age groups emphasize the need for age-tailored interventions that consider both physical limitations in older and mental health challenges in younger PWH.

In contrast to earlier studies [25] suggesting lower HRQoL among older PWH, our findings—consistent with a more recent study by Quach et al [38]—showed that PWH aged ≥50 years had higher SF-36 subdomain scores compared to their HIV-negative peers. However, overall PCS and MCS scores remained comparable between groups. This may reflect the positive influence of improved ART availability, including earlier ART initiation, simplified regimens, and enhanced long-term management [39]. The largely comparable prevalence of geriatric syndromes across groups may further explain these parallel HRQoL findings, as such syndromes are known to negatively impact quality of life [40]. This highlights the critical role of CGA and personalized care to address geriatric syndromes and thereby improve HRQoL in aging populations, both with and without HIV. Additionally, the relatively lower SF-36 subdomain scores in the HIV-negative group may be influenced by sociodemographic factors, such as a higher comorbidity burden and lower income, which was not analyzed in this study.

Several studies have demonstrated that ART modifications—such as transitioning to single-tablet regimens—can significantly improve HRQoL in PWH [12, 41]. However, to date, no studies have comprehensively examined the impact of multidisciplinary medication optimization on HRQoL specifically in older PWH. Our study showed significant improvements in both PCS and MCS scores at the 6-month follow-up after a multidisciplinary intervention, together with clinically meaningful improvements based on MCID. These findings suggest that multidisciplinary medication optimization may be a promising strategy for enhancing HRQoL in this population. However, due to the quasi-experimental design and the lack of a longitudinal control group, causality cannot be definitively established. The observed improvements may partly reflect alternative explanations, such as regression to the mean, the Hawthorne effect, or temporal changes in routine care. In addition, unmeasured confounding factors may have influenced the findings. Therefore, the results should be interpreted as time-related associations, with causal interpretations remaining limited due to the study design. The observed improvements may stem from the combined impact of several intervention components, including ART modifications, deprescribing of PIMs, and initiation of PPOs. These actions not only mitigate the risks associated with inappropriate prescribing but also promote alignment with contemporary standards in geriatric and HIV care.

This study has several limitations. First, the demographic and clinical characteristics, such as sex, age, and the number of co-medications, differed between older PWH and HIV-negative individuals, which may have introduced confounding effects. In addition, the comparator groups were not matched and may differ in unmeasured factors, such as socioeconomic characteristics, limiting the interpretability of baseline comparisons. Furthermore, due to the relatively small sample size and the number of potential confounders, adjusted analyses were not performed, which may have further limited the ability to account for confounding. Second, as no control group was included for the longitudinal analysis, the observed changes in HRQoL cannot be attributed solely to the intervention and may have been influenced by other factors, including increased clinical attention or temporal effects. Additionally, repeated measurements were only available for older PWH, limiting the use of advanced longitudinal analyses, such as mixed-effects models. Third, the 6-month follow-up period limits conclusions regarding the long-term sustainability of the multidisciplinary intervention and its impact on clinical outcomes.

In conclusion, this study demonstrated that a multidisciplinary approach is associated with reductions in inappropriate prescribing and improvements in HRQoL among older PWH. Given the complex and evolving healthcare needs of this aging population, the development of age-tailored, person-centered interventions is crucial to promote healthy aging and sustain long-term well-being. In this context, developing HIV-specific criteria and ensuring their integration into routine care may further improve medication optimization efforts. To achieve this, the establishment of comprehensive HIV care models and clinics should be prioritized to ensure coordinated, holistic, and high-quality care that addresses both medical and psychosocial dimensions of aging with HIV.

## Supplementary Material

ofag378_Supplementary_Data

## Supplementary Data

Supplementary materials are available at *Open Forum Infectious Diseases* online. Consisting of data provided by the authors to benefit the reader, the posted materials are not copyedited and are the sole responsibility of the authors, so questions or comments should be addressed to the corresponding author.
