# Supplementary material for: Multidisciplinary Care for Older People With HIV: Optimizing Medication and Enhancing Quality of Life
Source: Open Forum Infect Dis. 2026 Jun 22;13(7):ofag378. doi: 10.1093/ofid/ofag378 (PMC13329662; doi:10.1093/ofid/ofag378)
Supplement: ofag378_Supplementary_Data [file ofag378_supplementary_data.docx]

**Supplementary Table 1.** STOPP v2 criteria identified among PLWH aged ≥50 years.

| **Criteria section** | **Criteria** | **1st interview** | **2nd interview** |
| --- | --- | --- | --- |
| **Drug Indication** | Any drug prescribed without an evidence-based clinical indication. | 34 | 23 |
|  | Any drug prescribed beyond the recommended duration, where treatment duration is well defined. | 12 | - |
|  | Any duplicate drug class prescription | 2 | - |
| **Cardiovascular System** | Beta blocker with symptomatic bradycardia (<50/min), type II heart block or complete heart block. | 1 | - |
| **Gastrointestinal System** | Proton pump inhibitor (PPI) for uncomplicated peptic ulcer disease or erosive peptic oesophagitis at full therapeutic dosage for>8 weeks. | 1 | - |
| **Respiratory System** | Non-selective beta-blocker with a history of asthma requiring treatment. | 1 | 1 |
| **Urogenital System** | Selective alpha-1 selective alpha blockers in those with symptomatic orthostatic hypotension or micturition syncope. | 2 | - |
| **Endocrine System** | Thiazolidenediones in patients with heart failure | 1 | - |
| **Total** |  | **54** | **24** |

**Supplementary Table 2.** TIME-to-STOPP criteria identified among PLWH aged ≥50 years.

| **Criteria section** | **Criteria** | **1st interview** | **2nd interview** |
| --- | --- | --- | --- |
| **Cardiovascular System** | Use of rate-limiting therapy (e.g., beta-blocker, verapamil, diltiazem, digoxin) is inappropriate in patients with bradycardia, type II heart block, or complete heart block. | 1 | - |
|  | Use of vasodilatory antihypertensives and nitrates is inappropriate in patients with orthostatic hypotension. | 1 | - |
|  | Use of non-selective beta-blockers is inappropriate in patients with a history of asthma. | 1 | 1 |
| **Central Nervous System and Psychotropic Medications** | Use of paroxetine, fluoxetine, and fluvoxamine as first-line SSRIs is inappropriate in patients initiating SSRI therapy. | 1 | - |
|  | Continuous or prolonged use of medications for vertigo (e.g., betahistine, trimetazidine, dimenhydrinate) is inappropriate. | 1 | 1 |
|  | Use of citalopram at >20 mg/day or escitalopram at >10 mg/day is inappropriate. | 1 | 1 |
| **Gastrointestinal System** | Use of PPIs) at full therapeutic doses for more than 8–12 weeks in uncomplicated peptic ulcer disease or erosive esophagitis is inappropriate. | 1 | - |
|  | Use of PPIs due to polypharmacy is inappropriate. | 22 | 11 |
|  | Use of magnesium-containing laxatives or antacids is inappropriate in patients with GFR <30 mL/min/1.73 m². | 1 | - |
| **Musculoskeletal System and Analgesics** | Use of zoledronate, denosumab, or teriparatide without prior serum calcium assessment and adequate calcium/vitamin D supplementation is inappropriate. | 1 | - |
| **Urogenital System** | Use of non-uroselective alpha-1 blockers for the treatment of LUTS due to BPH is inappropriate in patients with orthostatic hypotension. | 1 | - |
| **Endocrine System** | Use of thiazolidinediones is inappropriate in patients with documented heart failure, a history of fractures, increased fracture risk, bladder cancer, or those receiving insulin therapy. | 1 | - |
|  | Use of SGLT-2 inhibitors is inappropriate in patients with GFR <45 mL/min/1.73 m². | 1 | - |
| **Antimuscarinic Anticholinergic Burden** | Use of medications with a high anticholinergic burden is inappropriate in patients with a history of falls, constipation, narrow-angle glaucoma, dementia, delirium, urinary retention, obstructive LUTS in men, or concomitant use of other high anticholinergic burden medications. | 1 | - |
| **Total** |  | 35 | 14 |

BPH: Benign Prostatic Hyperplasia, GFR: Glomerular Filtration Rate; LUTS: Lower Urinary Tract Symptoms; PPI: Proton Pump Inhibitor; SGLT: Sodium-Glucose Co-Transporter; SSRI: Selective Serotonin Reuptake Inhibitor.
